# Correlation between vitamin D and poor sleep status in restless legs syndrome

**DOI:** 10.3389/fendo.2022.994545

**Published:** 2022-09-15

**Authors:** Chaofan Geng, Zhenzhen Yang, Xiumei Kong, Pengfei Xu, Hongju Zhang

**Affiliations:** ^1^ Henan University People’s Hospital, Henan Provincial People’s Hospital, Zhengzhou, China; ^2^ Fuwai Central China Cardiovascular Hospital, Henan Provincial People’s Hospital, Zhengzhou, China; ^3^ Henan University Joint National Laboratory for Antibody Drug Engineering, Kaifeng, China; ^4^ Henan Provincial Chest Hospital, Zhengzhou, China; ^5^ Zhengzhou University People’s Hospital, Henan Provincial People’s Hospital, Zhengzhou, China

**Keywords:** Restless Legs Syndrome, vitamin D, sleep disorder, mechanism, case- control study

## Abstract

**Background:**

Restless Legs Syndrome (RLS) is closely related to poorer sleep quality. Vitamin D can regulate sleep regulation, cell proliferation, and differentiation. To measure whether vitamin D has predictive value for poor sleep quality in RLS was our aim in this study.

**Methods:**

To analyze the serum levels of 25-hydroxyvitamin D [25(OH)D] in 95 RLS patients. We used the Pittsburgh Sleep Quality Index (PSQI) to measure sleep quality. Subjects had been divided into a normal and poor-sleeper groups according to the PSQI score. Using correlation and regression analysis to explore underlying etiologies that affect sleep disorder in RLS patients.

**Results:**

Patients in the poor-sleeper group had significantly lower vitamin D levels in comparison to the normal group. The serum vitamin D levels were negative correlate with PSQI scores after adjusting for confounding factors. In addition, regression analysis showed that vitamin D could act as a predictor for sleep disorders in RLS patients (odds ratio [OR] = 0.008, *p* = 0.004). The area under the curve (AUC), cut-off value, sensitivity, and specificity of serum vitamin D was 0.967 (95% CI 0.935–0.998), 16.84 ng/ml, 87.5%, and 93.7% by receiver operating characteristic (ROC) analysis.

**Conclusion:**

Our study confirmed the relationship between poorer sleep quality and vitamin D in RLS. However, the causal relationship between vitamin D deficiency and RLS is currently inconclusive. The effect of vitamin D supplementation is needed to confirm as the therapeutic strategies for sleep disorders in RLS patients in future work.

## Introduction

Restless legs syndrome (RLS) is a sleep-related movement disorder (1), which manifests clinically as a strong desire to move both lower extremities, with symptoms often worsening at night (2). The global prevalence of RLS is 0.03% to 24.2% (3–5). RLS can be divided into two categories: primary and secondary forms. Additionally, previous studies had found that secondary RLS was associated with chronic renal failure (6), type 2 diabetes (7), etc. However, the pathogenesis of primary RLS has not been fully established, but genetic factors, impaired dopaminergic neuron function and brain iron deficiency are recognized to be associated with the development of primary RLS (8).

It has been reported that RLS patients also suffer from sleep disturbance and depression (9), which can strongly impact the quality of life (10). Additionally, previous studies had demonstrated that vitamin D can be able to regulate dopamine levels and its metabolites (11, 12). However, vitamin D deficiency is common in RLS patients (13, 14), which may participate pathogenesis and progress of the disease (13, 15). Furthermore, lower serum vitamin D level is related to worse sleep quality, and the severity of disease in RLS patients (12, 13), suggesting that vitamin D may have predictive value for pooter sleep quality in RLS patients. Indeed, vitamin D deficiency is associated with the risk of sleep disorders (16). Apart from that, vitamin D supplementation also is related to improving mood and quality of sleep (17). Therefore, based on these findings, we hypothesized that vitamin D can affect the sleep quality of RLS patients.

To our knowledge, the association of vitamin D with poorer sleep quality in RLS patients remains unclear. Therefore, in the present study, to investigate the predictive efficacy of vitamin D for poorer sleep quality in RLS patients was our aim, which may be contributing to the development of therapeutic strategies to diagnose and prevent RLS with poorer sleep quality.

## Materials and methods

### Research subjects

Our study was conducted from January 2018 to October 2021, including 95 patients with RLS who derived from the Henan Provincial People’s Hospital.

The Research Ethics Committee of Henan Provincial People’s Hospital approved our study. Additionally, all the participants signed the written informed consent forms.

### Inclusion criteria

Diagnosis of RLS according to International Classification of Sleep Disorder (ICSD-3) (18) and the International Restless Legs Syndrome Study Group (IRLSSG) (2, 19).

### Exclusion criteria

The following patients were excluded: 1) combined with endocrine and metabolic Diseases; 2) Patients with secondary RLS, for instance, Parkinson’s disease (PD), drug-induced factors (for instance: antidepressant treatment with SSRI, etc.), iron deficiency, chronic renal failure; 3) combined with other sleep disorders such as narcolepsy, or obstructive sleep apnea (OSA); 4) patients taking drugs that affected serum vitamin D levels; 5) history of mental disorders; 6) pregnant and lactating women.

### Clinical assessment

To measure the disease severity and quality of life in RLS, we used The International Restless Legs Scales (IRLS) (20), and The Restless Legs Syndrome Quality of Life Questionnaire (QoL-RLS) (21), respectively. Additionally, to measure depression and anxiety, the 24-item Hamilton Depression Rating Scale (HAMD_24_), and the 14-item Hamilton Anxiety Scale (HAMA_14_) were used.

### Sleep conditions and grouping

To measure the sleep quality, the Pittsburgh sleep quality index (PSQI) was used (22). Participants were classified into non-sleep disorders (normal, PSQI ≤ 5) and sleep disorders (poor-sleeper, PSQI > 5) groups, due to the PSQI score (22).

### Laboratory assessment

To measure serum 25-hydroxyvitamin D (25(OH)D) levels, we used the Enzyme-Linked Immunosorbent Assay (ELISA). Additionally, in our hospital, insufficient vitamin D level has been defined as < 20 ng/ml respectively.

### Statistical analysis

The SPSS Statics 22.0 software for analysis. Representation of non-normal date in interquartile range (M, P_25_, P_75_) and normal date in mean ± standard deviation (SD). The student’s-t test for normally distributed continuous data, while The Mann-Whitney U test for the non-normally date. Categorical data are expressed in amount (%). Spearman or Pearson to analyze the clinical characteristics and vitamin D in RLS. To determine the predictive value of serum vitamin D levels for RLS sleep disorders, receiver operating characteristic (ROC) curve analysis was used for evaluation. P < 0.05 was deemed statistically significant.

## Results

### Demographics and clinical characteristics

**Figure 1:**
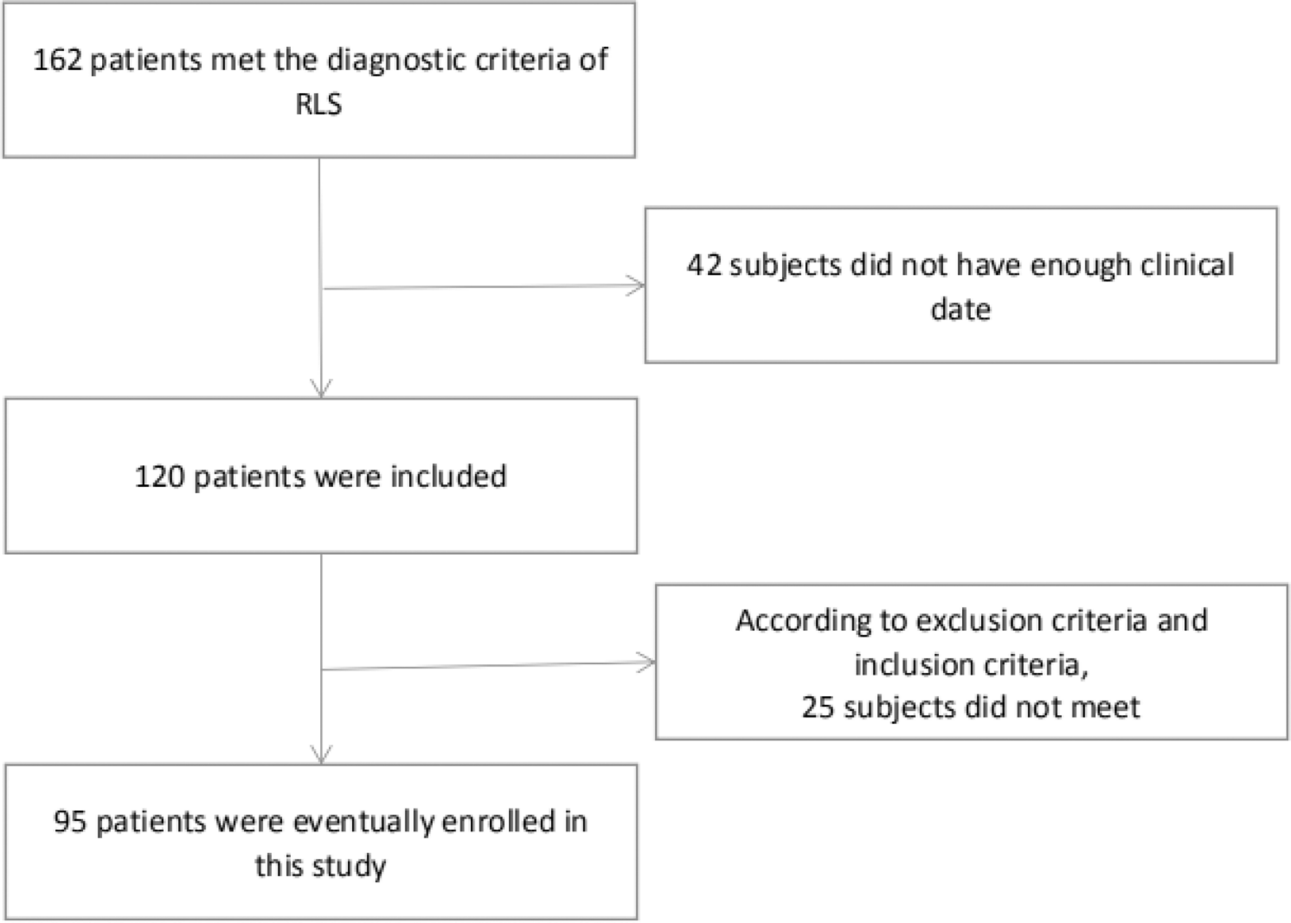
Flow chart of patient inclusion.

According to the PSQI score, subjects can be divided into the poor-sleeper group (n=63) and normal group (n=32). The baseline data of all subjects are summarized in **Table 1**. Compared to the normal group, the serum vitamin D level was lower in the poor-sleepers. In addition, no difference was found between both groups in age, sex, BMI, SBP, DBP, IRLS, QoL-RLS, disease duration, education duration, and sunlight exposure (*p* > 0.05) **(** **Figure 1** **)**.

**Table 1:** Demographic data and results of biochemical analyses.

	Poor-sleeper (n=63)	normal (n=32)	*P* value
Gender (male/female)	21/42	11/21	0.919
Age (years)	40.48 ± 6.08	40.63 ± 5.74	0.909
BMI (kg/m²)	23.65 ± 2.07	22.97 ± 1.62	0.110
SBP (mmHg)	125.29 ± 7.39	127.53 ± 9.01	0.197
DBP (mmHg)	78.95 ± 8.07	81.22 ± 9.06	0.218
Alcohol [n (%)]	13 (20.6%)	8 (22.5%)	0.961
Smoking [n (%)]	10 (15.9%)	8 (22.5%)	0.503
PSQI, points	12.92 ± 2.36	5.63 ± 1.21	**<0.001**
HAMA_14_, points	11.83 ± 3.61	9.56 ± 4.23	**0.008**
HAMD_24_, points	19.30 ± 1.74	15.72 ± 3.53	**<0.001**
IRLS, points	16.35 ± 5.28	15.00 ± 5.07	0.236
QoL-RLS, points	32.84 ± 3.70	31.56 ± 2.56	0.053
disease duration (year)	3.39 ± 0.48	3.55 ± 0.56	0.169
education duration (year)	10.86 ± 1.76	10.50 ± 1.50	0.329
Sunlight exposure (h/d)	8.74 ± 1.08	8.62 ± 1.18	0.618
Vitamin D (ng/ml)	14.44 ± 1.78	18.08 ± 1.07	**<0.001**

Poor-sleeper, sleep disorders (PSQI points > 7); normal, non-sleep disorders (PSQI points ≤ 7); BMI, body mass index; SBP, systolic blood pressure; DBP, diastolic blood pressure; PSQI, Pittsburgh Sleep Quality Index (PSQI) scale, HAMD_24,_ 24-item Hamilton Depression Rating Scale; HAMA_14_, 14-item Hamilton Anxiety Scale; IRLS, international restless legs syndrome rating scale; QoL-RLS, the Restless Legs Syndrome Quality of Life Questionnaire. Data are presented as mean ± standard deviation, or median (interquartile range) as appropriate. The differences were considered significant if p-value < 0.05. The bold values indicate the value of p < 0.05.

### Correlation analysis

Correlation analysis showed that serum vitamin D was negatively correlated with PSQI, HAMA_14_, HAMD_24_, and IRLS (*p* < 0.05). The partial correlation analysis was used to show that serum vitamin D was still positively associated with PSQI, HAMA_14_, HAMD_24_, and IRLS (*p* < 0.05), after adjustment for the factors of age, gender, BMI, disease duration, education duration, and sunlight exposure. There was no significant association between vitamin D levels and sex, age, BMI, disease duration, education duration, and sunlight exposure among the two groups (*p* > 0.05). The correlation analysis was shown in **Table 2**.

**Table 2:** Correlation between serum vitamin D and clinical characteristics of RLS.

Item	Vitamin D			
unadjusted				Adjusted*			
	r value	p value	r value	p value
Age	-0.108	0.296	–	–
Gender	-0.036	0.728	–	–
BMI	-0.057	0.586	–	–
PSQI, points	-0.740	**<0.001**	-0.782	**<0.001**
HAMA_14_, points	-0.239	**0.020**	-0.224	**0.035**
HAMD_24_, points	-0.423	**<0.001**	-0.424	**<0.001**
IRLS, points	-0.257	**0.012**	-0.217	**0.041**
QoL-RLS, points	0.006	0.952	0.038	0.724
disease duration (year)	-0.006	0.953	–	–
education duration (year)	-0.100	0.336	–	–
Sunlight exposure (h/d)	-0.155	0.133	–	–

*age, gender, BMI, disease duration, education duration, and sunlight exposure adjusted. The bold values indicate the value of p < 0.05.

### Regression analysis

Three regression models of regression analysis were shown in **Table 3**. In Model 1, serum vitamin D was an independent risk predictor for poorer sleep quality in RLS (*p* < 0.001; odds ratio=0.141; 95% confidence interval: 0.058–0.338). In Model 2, after further adjustment for age, sex and BMI, serum vitamin D was still an independent risk predictor for poorer sleep quality in RLS (*p* < 0.001; OR=0.046, 95%CI: 0.009). In Model 3, after adjustment for age, sex, BMI, disease duration, education duration and, sunlight exposure, serum vitamin D was also an independent risk predictor for poorer sleep quality in RLS (*p* = 0.004**;** OR=0.008, 95%CI:0.000,0.215).

**Table 3:** Regression analysis of serum vitamin D and the risk of developing poorer sleep quality in RLS.

	OR (95% CI)	*P* value
Model 1	0.141 (0.058,0.338)	**<0.001**
Model 2	0.046 (0.009,0.239)	**<0.001**
Model 3	0.008 (0.000,0.215)	**0.004**

OR, odds ratio; CI, confidence interval. Model 1: Unadjusted; Model 2: After adjusting for age, sex and BMI; Model 3: After adjustment for age, sex, BMI, disease duration, education duration and sunlight exposure. The bold values indicate the value of p < 0.05.

### ROC analysis

**Figure 2:**
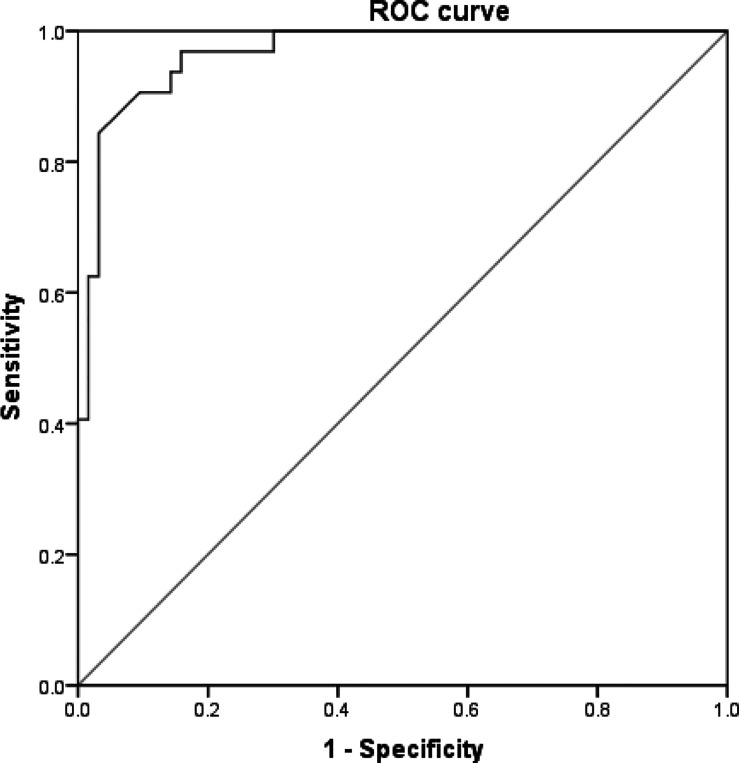
ROC curve of serum vitamin D for predicting poor sleep quality in RLS.

ROC analysis was performed to determine the predictive value of serum vitamin D for poorer sleep quality in RLS patients, which was shown in **Figure 2**. The area under the curve (AUC) was 0.967 (95% CI 0.935–0.998). In addition, the cut-off value of serum vitamin D was 16.84 ng/ml with a sensitivity of 87.5% and specificity of 93.7%.

## Discussion

### Summary of findings

Our study mainly found that serum vitamin D levels were significantly lower in poor-sleeper patients. Additionally, correlation regression analysis found that serum vitamin D can act as an independent predictor for sleep disorders in RLS. According to the ROC analysis, we can hypothesize that serum vitamin D may be a potential biomarker for predicting sleep disorders in RLS patients with high sensitivity and specificity.

### The pathogenesis of RLS

The exact pathophysiology of RLS is not fully established. Previous neuroimaging studies had demonstrated that dopaminergic dysfunction was associated with the pathogenesis of RLS (23–25). In addition, growing evidence suggests that iron deficiency plays an important role in the pathologic features and symptoms of RLS, influenced by a combination of environmental factors and genetic inheritance (26, 27). A growing body of evidence had demonstrated that iron can be a first-line treatment option for RLS (28–30). Moreover, there is also evidence of the link between iron deficiency and nigrostriatal dopamine dysfunction (31), which contributes to understanding the pathophysiology of the disease. However, brain iron deficiency associated with vitamin D has not been adequately studied.

### The connections between sleep quality, vitamin D and RLS

The prevalence of the poor-sleepers was found to be about 66% in our study, which is in line with most previous reports (9, 32). Previous studies confirmed that most RLS patients experience sleep disorders, mainly complaining of difficulty falling asleep (33). In addition, previous studies have reported that vitamin D deficiency was correlated with worse sleep quality in RLS patients (12, 13), and our results also suggested a statistically significant negative correlation between serum vitamin D level and PSQI points. Additionally, numerous clinical and epidemiological studies have shown that sleep disorder was a risk factor for depression in RLS patients (34, 35), which was consistent with our finding that the HAMA_14_ and HAMD_24_ values were significantly higher in the poor-sleepers of RLS.

Vitamin D is involved in the synthesis and release of neurotransmitters and acts as a neuroprotective role (36). Recent studies have found that vitamin D deficiency plays a role in the pathogenesis and development of RLS (13, 15). Previous studies have reported that the serum vitamin D level was correlated with the disease severity in RLS patients (12, 13), and our results also suggested a statistically significant negative correlation between serum vitamin D level and IRLS points. Previous studies confirmed that vitamin D deficiency was an independent risk factor associated with RLS (37). The incidence of RLS is significantly higher in patients with vitamin D deficiency (15). In contrast to these findings, Jimenez-Jimenez et al. (38) found that compared with HCs, serum levels of vitamin D were significantly higher in idiopathic RLS patients.

Currently, randomized clinical trials on vitamin D supplementation and RLS are not only small in the number of studies, but also have conflicting results (39, 40). In future studies, multi-center, large-sample clinical studies are needed to further illustrate the therapeutic effects of vitamin D supplementation on sleep quality and symptoms of RLS patients.

### The probable mechanism of vitamin D regulation of sleep

There are increasing evidence that vitamin D can regulate the circadian rhythm of sleep (41, 42). Several magnetic resonance imaging (MRI) studies had revealed an increased glutamic acid in the thalamus of RLS patients, which also was associated with sleep quality (43–45), providing insight into the role of glutamate and dopamine in regulating sleep–wake disorder (46). The pathogenesis of the regulatory role of vitamin D in sleep disorder with RLS patients is largely unknown. Interestingly, the results of a study revealed the death of dopaminergic neurons can be caused by decreased glutathione content (47). Furthermore, vitamin D can upregulate glial cell line-derived neurotrophic factor (GDNF) expression (48), which plays an essential role in the development of dopaminergic neuron (49). We hypothesized that vitamin D can induce and promote the growth, development and differentiation of dopaminergic neurons, which may affect mood and sleep-wake cycles.

Several studies have shown that physiological levels of vitamin D can inhibit the production of pro-inflammatory cytokines, such as interleukin-6 (IL-6) and IL-10 (50). A recent study on RLS has revealed increased circulating levels of inflammatory cytokines (51). A large number of studies have investigated the neuroinflammation was related to sleep disorders (46). The anti-inflammatory effects of vitamin D may be another explanation for vitamin D deficiency that can lead to sleep disorder in the disease. Additionally, the production of melatonin can be regulated by vitamin D (13), which is the primary sleep-regulating hormone (52). Several lines of evidence reveal that melatonin can benefit patients with sleep disorders related to various neurological disorders (53, 54). However, more research is needed on the more complex relationship between vitamin D and sleep disorders.

### Limitations and recommendations

There are some limitations. First, our study was unable to show a causal relationship between vitamin D deficiency and RLS and further prospective investigation is needed. Second, our study participants were only primary RLS patients. Finally, *in vitro* and *in vivo* studies on vitamin D development and prevention in RLS patients with sleep disorders are lacking.

## Conclusion

Our study is the first to confirm the relationship between vitamin D and poor sleep status in RLS, and it could act as a useful biomarker for the development of therapeutic strategies to diagnose and prevent RLS with poor sleep quality. To confirm the effect of vitamin D supplementation as the therapeutic strategy for sleep disorders in RLS patients is needed in a larger population in future work.

## Data availability statement

The original contributions presented in the study are included in the article/supplementary material. Further inquiries can be directed to the corresponding author.

## Ethics statement

The study was approved by the Research Ethics Committee of Henan Provincial People’s Hospital according to the principles of the Helsinki Declaration. The patients/participants provided their written informed consent to participate in this study. Written informed consent was obtained from the individual(s) for the publication of any potentially identifiable images or data included in this article.

## Author contributions

CG: wrote first draft and statistics. ZY, PX, and XK: statistics and data collection. HZ: conceptualization, resources and supervision. All authors approved the submitted version.

## Conflict of interest

The authors declare that the research was conducted in the absence of any commercial or financial relationships that could be construed as a potential conflict of interest.

## Publisher’s note

All claims expressed in this article are solely those of the authors and do not necessarily represent those of their affiliated organizations, or those of the publisher, the editors and the reviewers. Any product that may be evaluated in this article, or claim that may be made by its manufacturer, is not guaranteed or endorsed by the publisher.
